# Methods of Control of Fragments in Gunshot Wounds of the Jaws

**Published:** 1918-12

**Authors:** 


**Methods of Control of Fragments in Gunshot Wounds of the Jaws.** By H. P. Pickerill, M. D. M. D. S. *The Lancet*, September 7, 1918.

Different forces contrive to produce the displacements of the fragments after gunshot wounds of the jaws : (1) the direction of the missile; (2) its size; (3) its velocity; (4) muscular action; (5) fibro-cicatricial contraction.

*Muscular Action* : Nearly all jaw muscles, besides their elevating or depressing action, may exert some other actions in quite different directions, and these happen to be very exaggerated when the continuity of the mandible is broken. For instance, the *temporal* muscle, which has normally an upward and backward pull, also exerts an outward pull of 2 cm. on each side. The *Masseter* likewise is an elevator of the mandible, and will also pull a posterior fragment altogether forward and outward, and so on for all muscles of the jaws. The following considerations are of importance :

(*a*) Abnormally developed muscles, or muscles used exclusively after the wound, may produce some displacements different from those expected.

(*b*) The condition of the various groups of muscles when the wound is received has some effect upon the amount and direction of displacement. This will vary as the muscles are contracted or relaxed.

*Control of Fragments* : As a general principle the fragments should be reduced to a normal position and maintained there by means of some appliance acting in an opposite direction to the displacing forces. The author does not approve of the use either of the four-tail bandage or of the Hammond splint. He does not consider them as sufficient means of control.

*Control of the Posterior Fragment* : When this is edentulous some form of saddle can usually be fitted to a metal cap splint. The saddle can either be rigid, and reduction be accomplished forcibly under anesthetics, or attached by screws which will permit of gradual reduction.

When the fragment happens to be anterior to the masseter but posterior to the last molar, no sufficient horizontal ramus exists to which a saddle can be attached. The author thinks that the best method of control in that case is the following : the loose fragment is pulled into position forcibly and maintained there; an incision is made over the zygoma; a hole drilled through the latter and the coronoid process; and a long thin screw (3/4 in.) is passed through both. No movement of the upper fragment is possible.

*The Gutter-Splint* : It consists of a gutter shaped to the dental arch and filled with modelling composition. Two arms are attached to the gutter, which project out of the mouth and lie along the cheeks. The displacement of the fragments is reduced by digital pressure, the composition warmed and the splint pressed into place, the arms being held into position by means of a bandage.

*Fractures of the Neck of the Condyle and Coronoid Process* : In these cases, since the upper fragment cannot be controlled, the lower one must be placed in such a position as to secure good alignment. This may best be done by intermaxillary wiring, either by silver wires around the teeth, or by passing crossed silver wires through four holes, two of which are drilled in the upper jaw and two in the lower one.

*Fractures at the Symphysis* : Intermaxillary wiring proves useful in such cases, or bolting of the fragments with a sort of metal bolt passed through the lower border of the mandible posterior to the symphysis.

*The Control of Comminuted Fragments* : The complete remova of all fragments generally proves unnecessary, and in most cases a conservative line of treatment may be adopted. The control of all small fragments is, however, often rather difficult. The author has devised a sort of splint controlling both the teeth and the fragments. It consists of a cap fitting over the retained teeth with arms passing out of the mouth and under the mandible. To the ends of these arms is hinged a padded lever which, by being depressed by a screw anteriorly, is raised in an upward and foward direction posteriorly. In use, the teeth are pressed down into their sockets, the fragments are pressed upwards and are clamped between the cap and the pad with an increasing pressure.

This sort of splint does not immobilize the mandible and this proves most important, as function is the most efficient stimulus to bone production.

*Submandibular Splinting* : The appliance of the interdental splint method to the lower and external border of the mandible has been found to be useful in edentulous cases. Screws are inserted into the lower border of the mandible through incisions in non-infected areas. The heads of the screws then serve the same purpose as teeth for the fixation of a splint.

*Fibro-cicatricial Contraction* : This tends to produce fresh displacements, or to exaggerate old ones. It may be dealt with either surgically and rapidly, or gradually and mechanically. The author considers the first method more advisable whenever it is possible.

